# HitWalker2: visual analytics for precision medicine and beyond

**DOI:** 10.1093/bioinformatics/btv739

**Published:** 2015-12-26

**Authors:** Daniel Bottomly, Shannon K. McWeeney, Beth Wilmot

**Affiliations:** ^1^Knight Cancer Institute,; ^2^Oregon Clinical and Translational Research Institute and; ^3^Division of Bioinformatics and Computational Biology, Department of Medical Informatics and Clinical Epidemiology, Oregon Health and Science University, Portland OR 97239, USA

## Abstract

**Summary:** The lack of visualization frameworks to guide interpretation and facilitate discovery is a potential bottleneck for precision medicine, systems genetics and other studies. To address this we have developed an interactive, reproducible, web-based prioritization approach that builds on our earlier work. HitWalker2 is highly flexible and can utilize many data types and prioritization methods based upon available data and desired questions, allowing it to be utilized in a diverse range of studies such as cancer, infectious disease and psychiatric disorders.

**Availability and implementation:** Source code is freely available at https://github.com/biodev/HitWalker2 and implemented using Python/Django, Neo4j and Javascript (D3.js and jQuery). We support major open source browsers (e.g. Firefox and Chromium/Chrome).

**Contact:** wilmotb@ohsu.edu

**Supplementary information:** Supplementary data are available at *Bioinformatics* online. Additional information/instructions are available at https://github.com/biodev/HitWalker2/wiki

## 1 Introduction

Across domains, the need to integrate and prioritize genes or variants is a common theme—for therapeutic selection, as well as for mechanistic and perturbation studies. Network-context methods can facilitate the ranking of genes and associated genetic variants/mutations. For instance, some serve to rank the variant genes of individual subjects relative to orthogonal biological assay data (Bottomly *et al.*, 2013; Jia and Zhao, 2014) whereas others can be used for GWAS or QTL studies (Köhler *et al.*, 2008). As a whole, these approaches can integrate different data types and statically report results, but up to now have not focused on making the data ‘accessible’ with respect to discovery, interpretation and knowledge acquisition. To this end we developed HitWalker2, which is a highly customizable approach to both producing a ranked list of genes utilizing network and external information and exploring these results using graph-centric interactive visualizations. Details of the HitWalker2 workflow and framework itself can be found in the Supplementary Material.

## 2 Description of software

### 2.1 Overview

The original HitWalker R package provided a means to prioritize variants stored in an SQL database and display the results using a static image of a relevant subnetwork and a text summary that could be exported to an Excel document. Interaction with the program was via R syntax and as such it required a working R environment or access to a server that does and some familiarity with the language (or additional training time).

HitWalker2 contains all of the features of the original but additionally was designed as an approach to allow users to access meaningful aspects of their data through interactions with genes, subjects and groups as a series of graphs organized as panels. The vast majority of these interactions involve mouse clicks and dragging operations, a paradigm which bench scientists and clinicians will be familiar with.

HitWalker2 extends the static subnetwork image of HitWalker to allow user interactivity along multiple levels. This interactivity is not just due to relatively basic features such as reorganizing nodes and retrieving information about nodes. More importantly, it provides the ability to ask biologically meaningful questions with or without use of a prioritization approach.

### 2.2 Functionality

In the original Hitwalker framework, the key focus was on prioritizing genes in individual samples. We have extended this in HitWalker2 to now include: (i) summarization and subsetting of cohort-level phenotypic attributes, (ii) cohort-level identification of recurrent genes based on ‘hits’ (as defined by thresholding multiple biological assay results), (iii) identification of subset of cohort with the same hit results in a given set of genes, (iv) pathway-context for results (individual, subset and full cohort), (v) ability to group subjects and genes by specified relationships/queries, (vi) ability to export results via CSV/PDF and (vii) fully interactive web-based visualization platform.

## 3 Applications

### 3.1 Overview

We created a base database for human samples consisting of high confidence STRING interactions (Jensen *et al.*, 2009), pathways from Pathway Commons (Cerami *et al.*, 2011) and gene symbol mapping information from NCBI (Maglott *et al.*, 2005). This database is available at our Github wiki along with steps used in its creation.

#### 3.1.1 Precision medicine use case

**Fig. 1:**
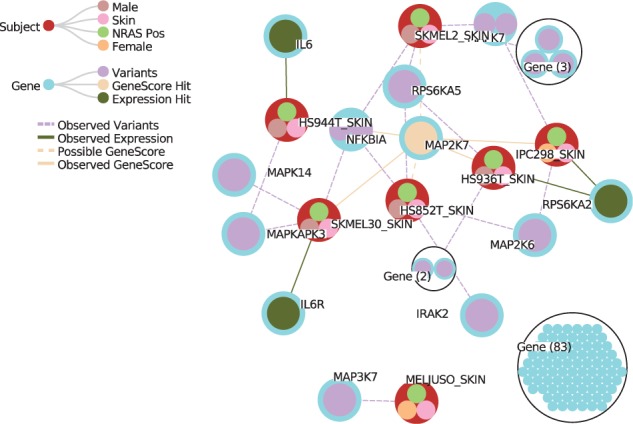
NRAS mutant skin cell lines mutations in the TLR3 Cascade pathway. The set of cell lines with NRAS mutations in the exome data was first retrieved and subsetted to only those cell lines derived from skin. Drug treatment data were used to identify MAPK7 as the most frequently perturbed gene target (GeneScore). The user can then explore the mutational burden of the pathways containing MAPK7, in this example, the Toll-Like Receptor 3 Cascade pathway from Reactome

One of the major translational challenges is target classification and prioritization in the clinical setting (Andre *et al.*, 2014). In this use case, the goal is to rank the cancer variants relative to expression and drug sensitivity that could guide therapeutic selection (Fig. 1). To illustrate this use case, we utilize cancer expression, variant and drug data from the Cancer Cell Line Encyclopedia (Barretina *et al.*, 2012). An expanded demo and walk-through of this example is provided on the Github wiki. We note that Hitwalker2 provides context to allow the user to move beyond individualized prioritization. For the HepG2 example, we have identified a prioritized gene set derived from the corresponding inhibitor assay results. We can then determine whether other samples or members of the cohort have mutations in the same genes (Supplementary Fig. S1). The interactive panels allow both individual and cohort level summaries. For example, at the cohort level, one can rapidly determine the most recurrently mutated genes in the cohort or for a selected subset of the cohort. Any overexpressed genes or drug sensitivity hits will automatically be displayed as part of the result. Pathways containing interesting genes can then be visualized (Supplementary Fig. S2). Exporting these results can be done via images of the graph panel(s) as well as text files.

#### 3.1.2 GWAS evidence-based visualization use case

ADHD GWAS results (Psychiatric Genetic Consortium; http://www.med.unc.edu/pgc/) were combined with study genotypes (unpublished data, JN and BW) and DNA methylation *P*-values (Wilmot *et al.*, 2015). In addition to top SNPs identified from a single GWAS study, it is useful to combine GWAS from multiple data sets and additional data types to strengthen the support for biologically important SNPs. For this use case, the focus is on rapidly identifying candidate genes with support across multiple studies and data types (Supplementary Fig. S3). *P*-values from a case/control meta-analysis of four Attention Deficit Hyperactivity Disorder datasets were used to compare with SNPs from a candidate gene study. This use case could also allow identification of inconsistencies across studies and provide network-context to GWAS results from both a patient and cohort perspective.

## 4 Discussion

HitWalker2 provides a visual, reproducible and flexible framework for prioritization that is applicable to a large number of clinical, translational and basic science use cases. The interactive framework facilitates discovery and guide interpretation in a robust and scalable way. This is timely given the growing recognition for more emphasis on human–data interactions.

The HitWalker2 framework is datatype agnostic and can include any type of experiment/assay which at the end suggests an effect on a gene such as variant/mutation data, copy number variation, expression as well as siRNA or drug screens.

## Supplementary Material

Supplementary Data
