# Supplementary material for: HitWalker2: visual analytics for precision medicine and beyond
Source: Bioinformatics. 2015 Dec 26;32(8):1253–5. doi: 10.1093/bioinformatics/btv739 (PMC4824131; doi:10.1093/bioinformatics/btv739)
Supplement: Supplementary Data [file supp_32_8_1253__index.html]

HitWalker2: Visual analytics for precision medicine and beyond — HitWalker2: visual analytics for precision medicine and beyond — HitWalker2: visual analytics for precision medicine and beyond — Supplementary Data 

# HitWalker2: visual analytics for precision medicine and beyond

## Supplementary Data

files

- Supplementary Data - pdf file
