# Supplementary material for: HitWalker2: visual analytics for precision medicine and beyond
Source: Bioinformatics. 2015 Dec 26;32(8):1253–5. doi: 10.1093/bioinformatics/btv739 (PMC4824131; doi:10.1093/bioinformatics/btv739)
Supplement: Supplementary Data [file supp_btv739_HitWalker2_supplemental_r1_v3.pdf]

1 Supplemental Description of Software

1.1 Workflow

The main workflow utilized by HitWalker2 is for the precision medicine use case utilizing the random walk with restarts algorithm. Users choose a single subject/sample, modify any algorithm parameters and can export the ranked list as a table or display the results in our panel view. The panel view allows users to query the data both visually and with HTML based menus. Prioritization is not a requirement to get to the panel view. A given user can also choose a single subject based on name or multiple subjects based on attribute assigned when data is loaded such as tissue or disease status.

1.2 Datatypes

HitWalker2 currently defines two types of objects that it uses for visualization/querying: genes and subjects. There are three classes of datatypes for genes in HitWalker2 that differ based on how they are used with respect to the prioritization algorithm. The 'Query' datatype is what is used to determine which genes should be prioritized. The typical example would be variant or mutation data. 'Hit' and 'Overlay' datatypes are both dichotomous (e.g. significant versus non-significant) and differ only in whether they are used for prioritization. The results from these datatypes become attributes for the gene nodes as well as edges as is described below. Subjects on the other hand can be assigned any number of categorical attributes though it is best to limit them to only those most relevant to the end user.

Hitwalker is "datatype agnostic" and supportive of diverse annotation sources and data standards for reporting. This is because depending on the use case, there is the need to utilize different public sources of annotation or incorporate different data standards. For clinical use cases, this will support the use of public repositories such as ClinVar (Landrum *et al.*, 2014) or OMIM (http://omim.org) as well as data standards such as ACMG and others. The Ensembl Variant Effect Predictor (McLaren *et al.*, 2010) can be added to a workflow with Hitwalker2 to annotate the provided variants with the desired information. This data can be output as part of the routine use of HitWalker2. Additionally, custom gene sets, say derived from specific diseases in OMIM, can be added to the database used to prioritize genes or simply to highlight phenotypic association with a particular gene.

1.3 Visualization Approach

All HitWalker2 visualizations utilize a panel system and each panel consists of one or more nodes and edges represented as the traditional circles and lines but with some differences.

Edges: In addition to genes our subjects are also represented as nodes which means that we also define subject to gene relationships in addition to the typical gene to gene relationships.

Nodes: Our nodes are hierarchical containers that can contain attributes distinguished by color. Attributes can be defined per subject such as gender, ethnicity or disease type or in the case of genes indicate a distinct type of 'Hit' was seen for a subject. In this manner the gene attributes inform the user prominently that the gene in question has a hit in one or more subjects while the subject to gene relationships indicate which subjects have that particular hit.

MetaNodes: In addition we also define a structure consisting of nodes containing other nodes, which is similar to containers previously used to represent protein complexes (Hu *et al.*, 2007). Nodes/metanodes can be moved within a panel to reorganize an image or between panels to indicate that a new panel should be formed with the union of the two sets of nodes grouped into metanodes by the distinct relationship types.

In addition to the operations defined for the nodes and edges HTML based menus are used to facilitate searching and querying. All visualizations are SVG graphics formed using D3.js with additional supporting javascript and jQuery code.

1.4 Database and Web Framework

Python, specifically the Django web framework is used to provide web content. We utilize Neo4j as the main database as it provides a great deal more flexibility and has similar performance to a traditional SQL database in our hands for the desired queries. An R package that is bundled with the source code simplifies the data loading process and allows for consistent representation of common bioinformatics objects implemented in Bioconductor such as the ExpressionSet (Gentleman *et al.*, 2004).

Although the HitWalker2 software stack can be implemented on any given server, we provide a provisioned virtual machine installation via Vagrant that simplifies and streamlines the process dramatically.

2 Supplemental Figures

A.)

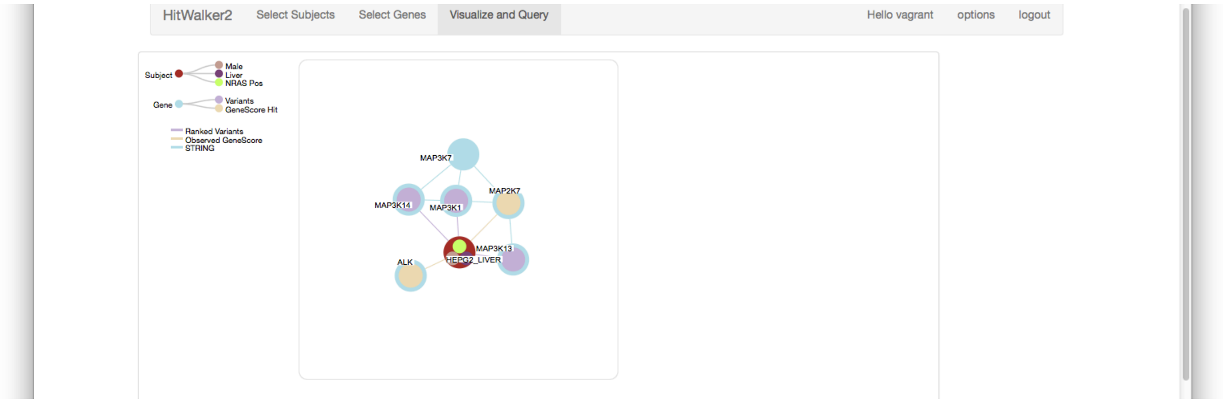
HitWalker2
Select Subjects
Select Genes
Visualize and Query
Hello vagrant
options
logout
Subject
Male
Liver
NRAS Pos
Gene
Variants
GeneScore Hit
Ranked Variants
Observed GeneScore
STRING
MAP3K7
MAP3K14
MAP3K1
MAP2K7
MAP3K13
HEPG2_LIVER
ALK

B.)

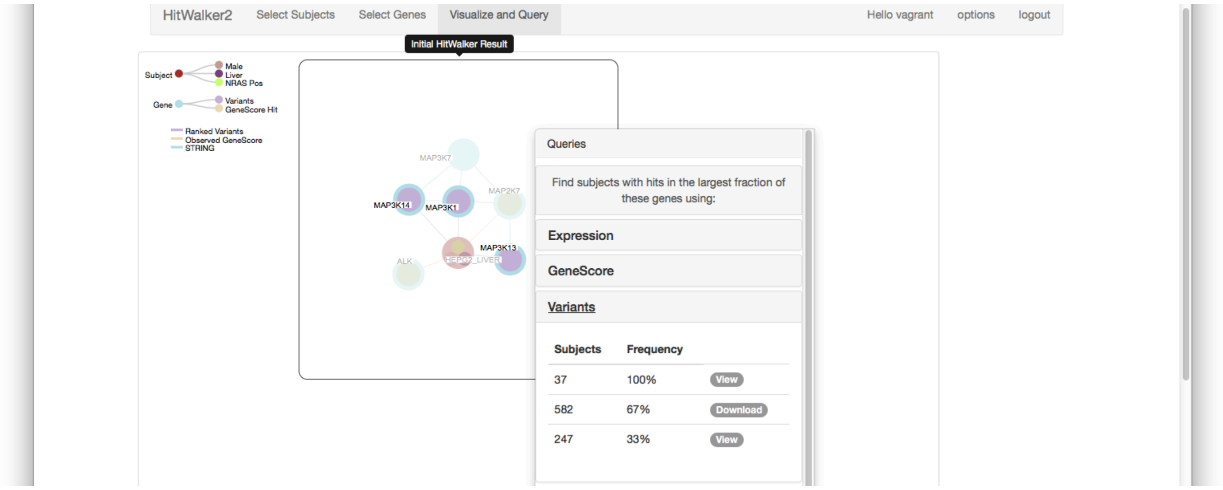
HitWalker2
Select Subjects
Select Genes
Visualize and Query
Hello vagrant
options
logout
Initial HitWalker Result
Subject
Male
Liver
NRAS Pos
Gene
Variants
GeneScore Hit
Ranked Variants
Observed GeneScore
STRING
MAP3K7
MAP3K14
MAP3K1
MAP2K7
MAP3K13
HEPG2_LIVER
ALK
Queries
Find subjects with hits in the largest fraction of these genes using:
Expression
GeneScore
Variants
Subjects
Frequency
37
100%
View
582
67%
Download
247
33%
View

C.)

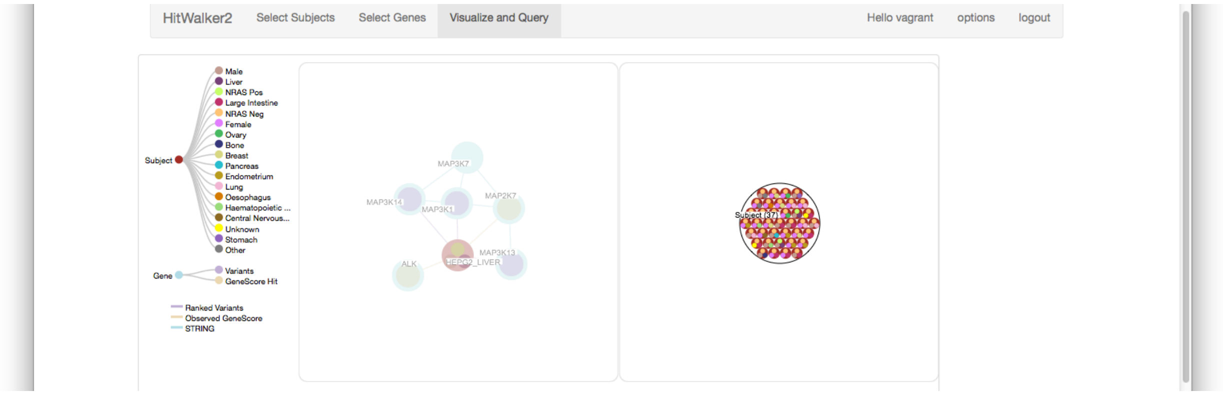
HitWalker2
Select Subjects
Select Genes
Visualize and Query
Hello vagrant
options
logout
Subject
Male
Liver
NRAS Pos
Large Intestine
NRAS Neg
Female
Ovary
Bone
Breast
Pancreas
Endometrium
Lung
Oesophagus
Haematopoietic ...
Central Nervous...
Unknown
Stomach
Other
Gene
Variants
GeneScore Hit
Ranked Variants
Observed GeneScore
STRING
MAP3K7
MAP3K14
MAP3K1
MAP2K7
MAP3K13
HEPG2_LIVER
ALK

D.)

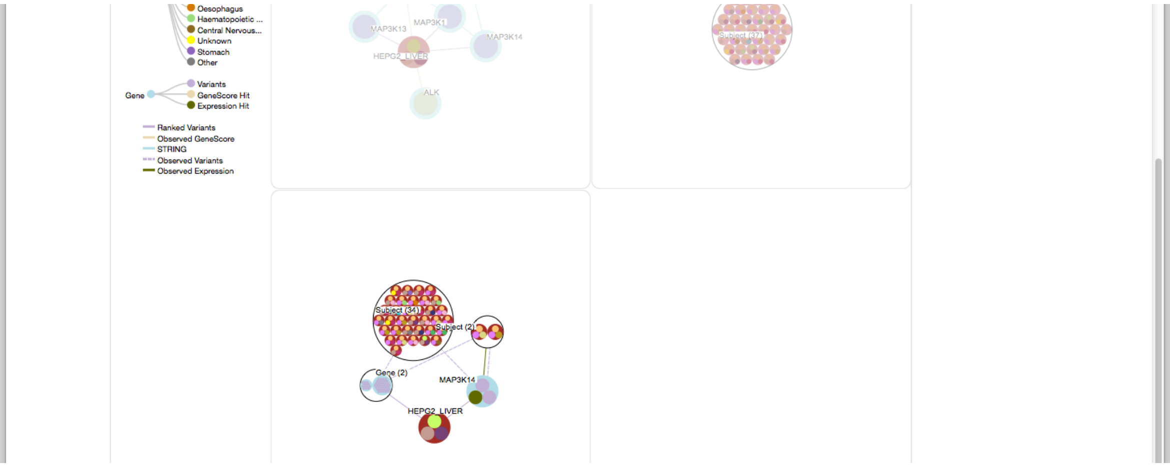

Fig S1. Finding cell lines with mutations in top ranked variants for the CCLE Hep G2 cell line. The prioritization algorithm with default parameters was applied to the mutations and drug sensitivity results for the Hep G2 cell line (see https://github.com/biodev/HitWalker2/wiki/ccle_example for more information on the setup). A.) A visualization of a subnetwork involving the mutation genes and drug sensitivity hits (GeneScore datatype) is provided after the prioritization procedure. B.) By selecting and right clicking on the three genes with mutations a menu is displayed which allows the user to see how frequent one or more of the three mutated genes is in the remainder of the CCLE cohort. For instance there are 37 cell lines which have at least one mutation in all three genes. C.) By clicking on 'view' a metanode representing all 37 subjects is shown in a new panel. D.) A new panel is generated by dragging over the 3 genes from the original prioritization result to the second panel with the subject metanode. The new panel contains both the 37 subject nodes and the 3 gene nodes grouped into nodes/metanodes by the type of relationship. Two of the subjects have up-regulated expression in MAP3K14 and observed variants in the other two genes while 35 of the subjects just simply have observed variants. Note here that 'Ranked_Variant' for the Hep G2 subject is distinguished from observed variants amongst the other 36.

A.)

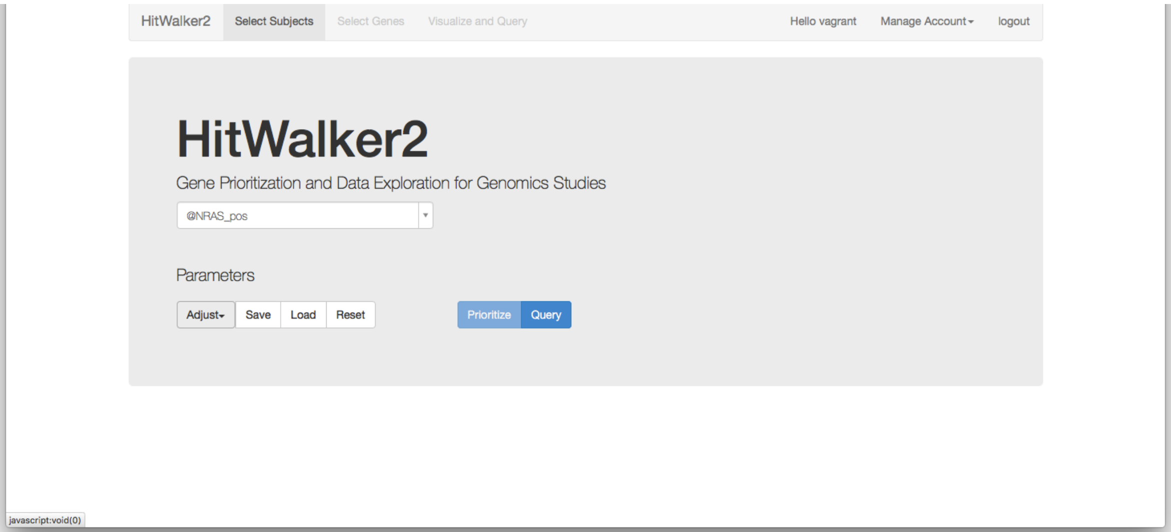

B.)

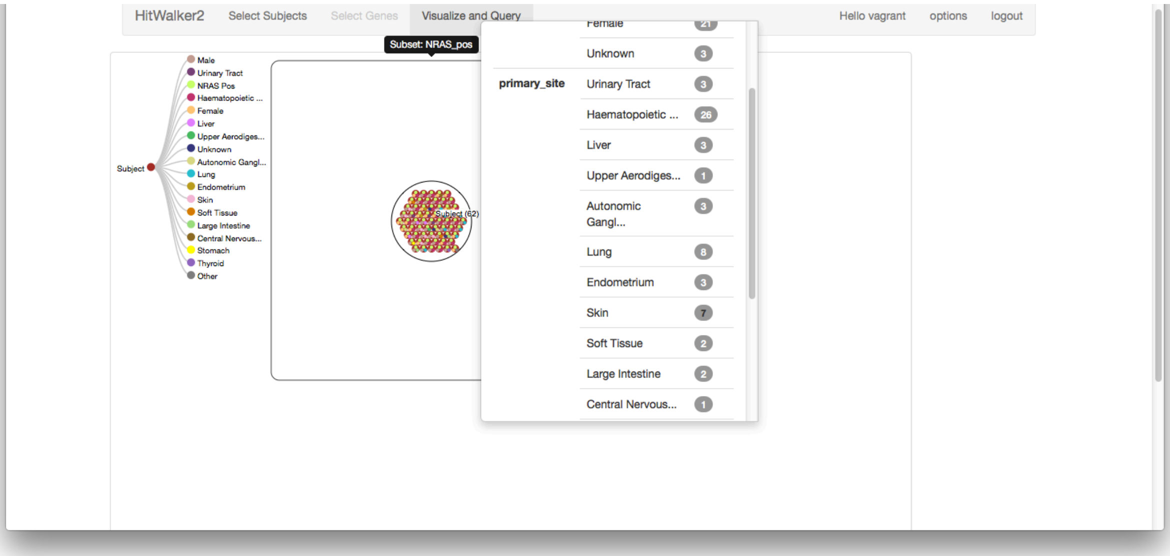
HitWalker2
Select Subjects
Select Genes
Visualize and Query
Hello vagrant
options
logout
Subset: NRAS_pos
Subject
Male
Urinary Tract
NRAS Pos
Haematopoietic ...
Female
Liver
Upper Aerodiges...
Unknown
Autonomic Gangl...
Lung
Endometrium
Skin
Soft Tissue
Large Intestine
Central Nervous...
Stomach
Thyroid
Other
Subject (62)
Unknown 3
primary_site
Urinary Tract 3
Haematopoietic ... 26
Liver 3
Upper Aerodiges... 1
Autonomic Gangl... 3
Lung 8
Endometrium 3
Skin 7
Soft Tissue 2
Large Intestine 2
Central Nervous... 1

C.)

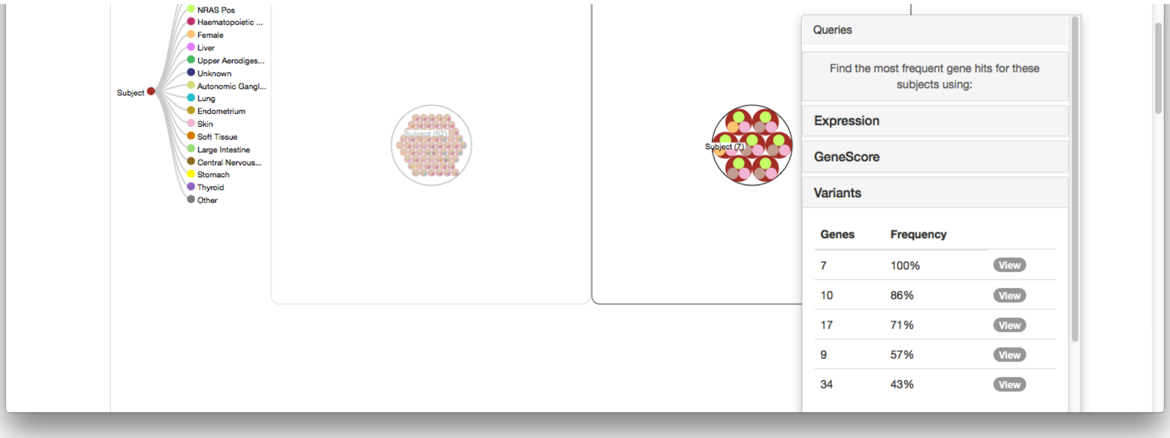
Subject
NRAS Pos
Haematopoietic ...
Female
Liver
Upper Aerodiges...
Unknown
Autonomic Gangl...
Lung
Endometrium
Skin
Soft Tissue
Large Intestine
Central Nervous...
Stomach
Thyroid
Other
Subject (7)
Queries
Find the most frequent gene hits for these subjects using:
Expression
GeneScore
Variants
Genes
Frequency
7 100% View
10 86% View
17 71% View
9 57% View
34 43% View

D.)

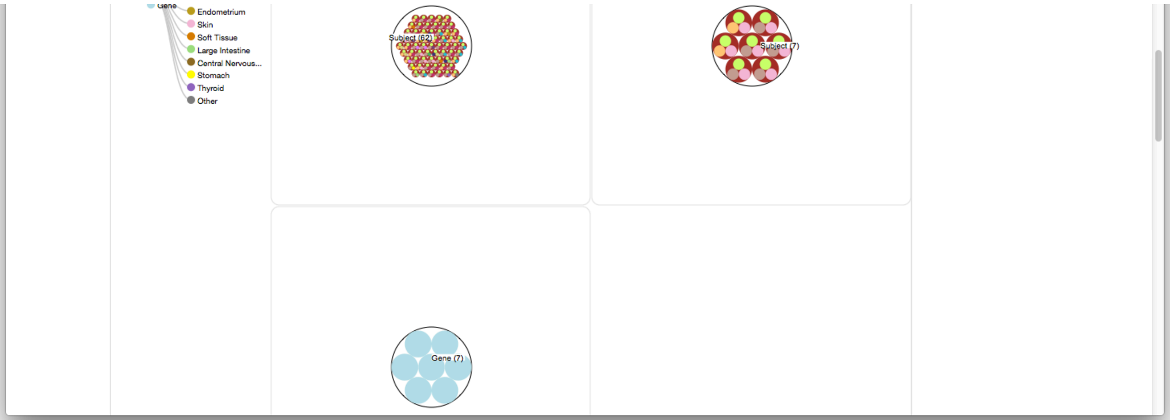
Endometrium
Skin
Soft Tissue
Large Intestine
Central Nervous...
Stomach
Thyroid
Other
Subject (62)
Subject (7)
Gene (7)

E.)

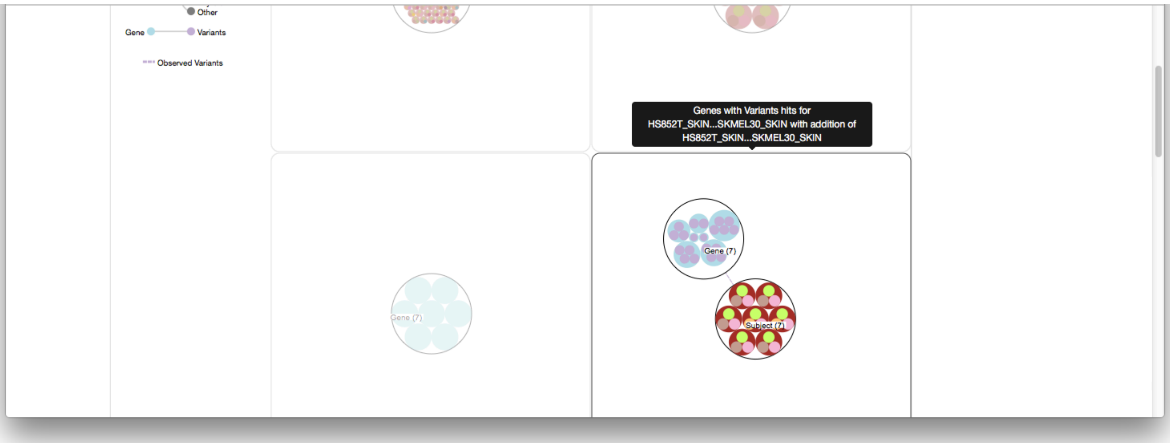
Other
Gene
Variants
Observed Variants
Genes with Variants hits for HS852T_SKIN...SKMEL30_SKIN with addition of HS852T_SKIN...SKMEL30_SKIN
Gene (7)
Gene (7)
Subject (7)

F.)

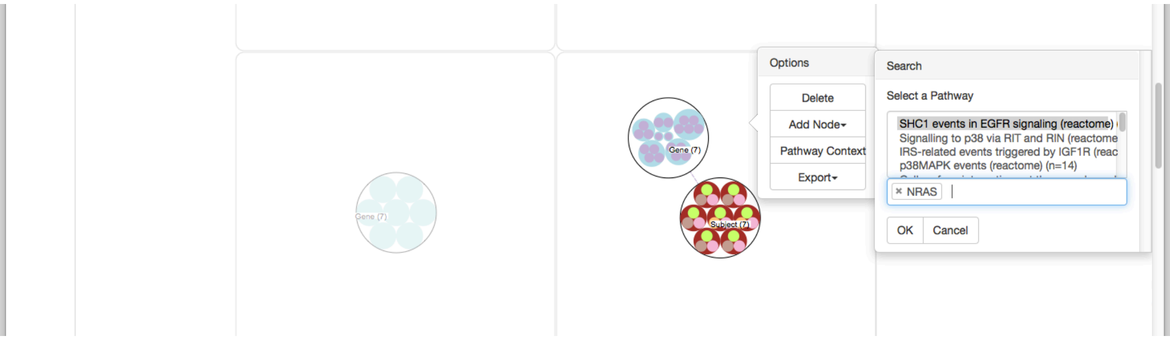
Options
Delete
Add Node
Pathway Context
Export
Search
Select a Pathway
SHC1 events in EGFR signaling (reactome)
Signalling to p38 via RIT and RIN (reactome
IRS-related events triggered by IGF1R (reac
p38MAPK events (reactome) (n=14)
NRAS
OK
Cancel
Gene (7)
Gene (7)
Subject (7)

G.)

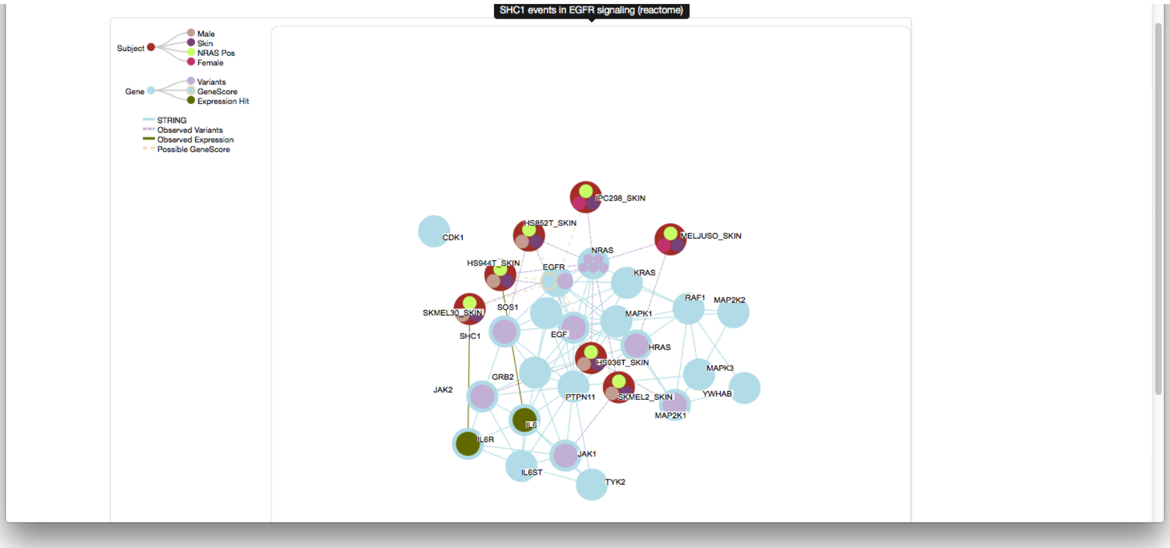

Fig S2. Accessing pathways containing recurrently mutated genes in the NRAS mutant skin cancer cell lines. The CCLE data was loaded and configured as shown in: https://github.com/biodev/HitWalker2/wiki/ccle_example. A.) First, the NRAS mutant cell lines were specified via the custom attribute ‘NRAS_pos’ defined for them. B.) On clicking ‘Query’ a metanode containing this subgroup is produced. By right clicking on the metanode, a new menu is displayed and the skin cancer cell lines can be chosen by clicking on the appropriate badge under the ‘Count’ column. C.) This generates a new panel and metanode containing the skin cancer cell line subset of the NRAS mutant cell lines. By selecting this metanode and right clicking, the frequency menu is brought up which, upon selecting the ‘Variants’ tab displays the number of genes and the frequency of samples with a mutation in those genes in the current metanode. D.) By clicking ‘View’ on the first entry a corresponding metanode of genes is produced. E.) By dragging into this new panel the metanode of NRAS mutant skin cancer cell lines, one can verify that all the cell lines have this mutation and that none of them are over-expressed or contain a drug sensitivity hit. F.) Right-clicking on the panel itself and clicking ‘Pathway Context’ allows the pathways containing these genes to be explored. G.) Once a pathway is selected it is visualized in a new panel along with the subject nodes and any mutations, over-expression or drug hits.

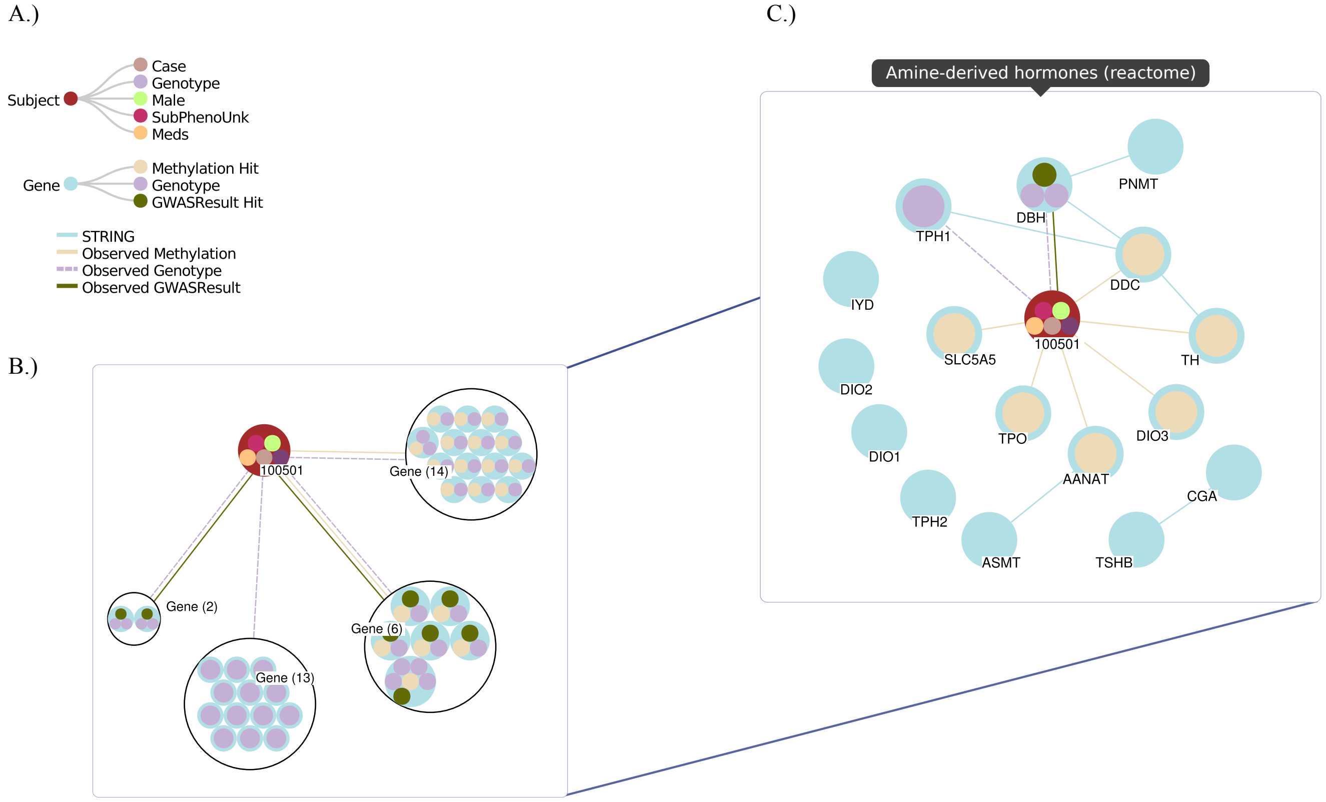

Figure S3. Augmenting study data with other public data sets to guide prioritization. The query algorithm was used to identify genes and pathways that have support from multiple sources. A) Panel overview and color coding. B) Visualization of one ADHD subject (red circle) as a representative of a study cohort that on which candidate genotyping (purple circles) and genome-wide methylation (beige circles) was performed. Genes that were nominally significant (unadjusted $p<0.05$) for both candidate genotypes and differential methylation between ADHD and controls were selected. Genome-wide association p-values (green circles) from an ADHD public meta-analysis (Neale, et al, 2010) were overlayed onto the study cohort data. Differences between ADHD and controls were supported by all three data types for six genes. C) Pathway membership for each of the six genes was examined Suppl. Figure 2 F. Pathway context of one of the candidate genes (DBH) is shown. It was noted that 37.5% of this pathway was differentially methylated between ADHD and control subjects.

3 References

Gentleman,R.C. et al. (2004) Bioconductor: open software development for computational biology and bioinformatics. *Genome Biol.*, 5, R80.

Hu,Z. et al. (2007) Towards zoomable multidimensional maps of the cell. *Nat Biotech*, 25, 547–554.

Landrum,M.J. et al. (2014) ClinVar: public archive of relationships among sequence variation and human phenotype. *Nucleic Acids Res.*, 42, D980–985.

McLaren,W. et al. (2010) Deriving the consequences of genomic variants with the Ensembl API and SNP Effect Predictor. *Bioinformatics*, 26, 2069–2070.

Neale,B.M. et al. (2010) Meta-Analysis of Genome-Wide Association Studies of Attention-Deficit/Hyperactivity Disorder. *J. Am. Acad. Child Adolesc. Psychiatry*, 49, 884–897.
